# General Nursing Council for Scotland

**Published:** 1920-06-26

**Authors:** 


### General Nursing Council for Scotland.

### SYNOPSIS OF DRAFT RULES.

The Chairman and Vice-Chairman to be elected annually and hold office for a year or until resignation, but to be eligible for re-election. At least twelve ordinary meetings of the Council to be held every year. The Registrar to keep the minutes and to be responsible for all arrangements relating to meetings of the Council and Committees. Seven days' notice to be given before meetings. Any relative documents and a copy of the minutes of the previous meeting to be sent with the notice. No business not on the agenda to be taken except by permission of the Chairman. Extraordinary meetings of the Council may be convened by the Chairman, or on the requisition of six members, stating the business they desire to have brought before the Council. Seven members to constitute a quorum. If after fifteen minutes' grace there is no quorum, the meeting to adjourn, and at the adjourned meeting the quorum to be five. In the absence of both Chairman and Vice-Chairman the meeting to appoint their own Chairman with full powers. The Chairman to have both a casting vote and a deliberative vote. At adjourned meetings no business to be transacted except what was left unfinished on the previous occasion. Council to appoint such Committees as they shall think fit and delegate thereto any of their powers and duties, provided that the powers and duties delegated to each Committee and its term of office and quorum be laid down definitely by the Council; that every Committee shall report its proceedings to the Council as directed; that no expenditure be incurred by a Committee without the sanction of the Council. Persons with special knowledge, not members of the Council, may be appointed to sit on any except the Finance Committee. The Council to have power to prescribe bye-laws for the procedure of Committees and for their convening.

All accounts to be examined by the Registrar and laid by him before the Finance Committee, who shall report them to the Council; all accounts to be initialled for payment by a member of the Finance Committee. All cheques for payment of money to be signed by a member of the Finance Committee and countersigned by the Registrar. A statement of the Council's financial operations in the current financial year up to the date of the previous meeting, showing the balance for the time being, to be presented by the Registrar at every ordinary Council meeting. All minutes, registers, and records to be open to the inspection of members of the Council during the Registrar's business hours.

